# Personalized Care in Advance Care Planning with Cancer and Chronic Progressive Diseases Using the Go Wish Game

**DOI:** 10.3390/jpm15050180

**Published:** 2025-04-30

**Authors:** Sara Alquati, Marta Perin, Simona Sacchi, Ludovica De Panfilis, Silvia Tanzi

**Affiliations:** 1 Palliative Care Unit, Azienda USL-IRCCS di Reggio Emilia, 42121 Reggio Emilia, Italy; sara.alquati@ausl.re.it (S.A.); simona.sacchi@ausl.re.it (S.S.); silvia.tanzi@ausl.re.it (S.T.); 2 Legal Medicine and Bioethics, Azienda USL-IRCCS di Reggio Emilia, 42121 Reggio Emilia, Italy; 3 Department of Medical and Surgical Sciences, Alma Mater Studiorum-University of Bologna, 40126 Bologna, Italy; ludovica.depanfilis@unibo.it; 4 IRCCS Azienda Ospedaliero-Universitaria di Bologna, 40138 Bologna, Italy

**Keywords:** advance care planning, end-of-life care, Go Wish Game, personalized medicine, cancer disease, neurological disease

## Abstract

**Background:** The Go Wish Game (GWG) is a card game designed to ease Advance Care Planning (ACP) discussions. It helps patients to consider their values and priorities, and to share them with families and healthcare professionals (HPs). Despite a general appreciation of the GWG, mechanisms related to its implementation have been poorly investigated. **Objective**: to determine optimal strategies for integration of the GWG into clinical practice and to investigate the experiences of a Palliative Care Unit (PCU) trained in the use of the GWG. **Methods**: We performed a descriptive qualitative study. Data were collected through a focus group (FG) and we followed the thematic analysis. The PCU’s team described their experience using the GWG focusing on 15 patients (9 oncological, 6 non-oncological) patients, managed by the PCU. **Results**: Our data revealed five main themes, along with their subthemes: (1) personalizing the GWG proposal; (2) the role of the caregiver; (3) organizational aspects; (4) meaning of the GWG in clinical practice, and (5) dealing with patient’s priorities. Comparison of real-life cases has led to the identification of specific facilitators and barriers that can hinder or promote the use of the GWG for personalized medicine. **Conclusions**: Findings suggest that certain aspects still require attention in GWG implementation, particularly regarding the training and competencies (communicative, relational, ethical) of the healthcare professionals, and the process leading to the proposal of the GWG to the patient.

## 1. Introduction

A personalized care approach is based on a care plan tailored to patient priorities and values, and it is fundamental in providing high-quality, patient-centered care. In line with the shared and ethical decision-making approach, Advance Care Planning (ACP) empowers patients to define their values, life goals, and medical preferences, supporting discussions with healthcare professionals (HPs) and family members concerning future care [1,2].

ACP, through active conversations on care preferences and end-of-life (EoL) issues [3], emphasizes the importance of patient autonomy in healthcare. This approach aims to align evidence-based practice and person-centered care, focusing on decision-making [3,4]. ACP improves patient–clinician communication, reduces unwanted hospital admissions, increases the use of palliative care, and enhances patient satisfaction and quality of life [5]. Despite the positive outcomes highlighted by the literature, the implementation of ACP presented several challenges depending on both patients’ and HPs’ difficulties in approaching ACP content [5,6].

Patients might find advance care planning (ACP) emotionally difficult and overwhelming because it involves facing the concept of death and organizing future medical care [7,8]. From the HPs’ perspective, the reluctance to discuss potential future deterioration with patients, the fear of diminishing hope, and the insufficient knowledge, skills, confidence, and time, constitute the most prevalent barriers to ACP [3,9]. Additionally, the literature highlights concerns affecting HPs’ views on timely EoL shared decision-making, especially the challenge of determining when to initiate the ACP conversation [4]. Therefore, it is crucial to identify effective tools that enhance and facilitate discussions on ACP.

The Go Wish Game (GWG) is a promising practical instrument, developed in 2005 by an American Association called Coda Alliance, to ease ACP conversations [10]. It provides a simple tool, composed of 35 cards, to identify individual priorities. The GWG has been used in different settings of care for several illnesses [11]. The cards address several themes, such as symptom control, autonomy, communication, and family issues as well as practical, financial, and spiritual needs. We recently translated and culturally adapted the GWG to the Italian context [12]. Findings from our study show that the use of the GWG is welcomed as a first step in clarifying values and personal thoughts regarding EoL issues. However, several challenges and barriers affect the use and application of the GWG [13]. They are mainly related to the following four domains: patients (such as patients’ resistance to discuss EoL issues, exacerbated by a lack of clarity about their priorities or emotional fragility in engaging in the discussion; clinical conditions; perception of their prognosis; and the stigma associated with death); HPs (lack of training in communication and empathy; lack of time and adequate support); family (difficult acceptance of patient’s condition, taboo surrounding death); and institutional context (lack of clear policies and organizational support; struggle to adapt the tool to the needs and workflows of the clinical setting) [13].

Despite the increasing need to understand optimal timing to propose and play the GWG, and the characteristics of the patients and further issues in terms of barriers/facilitators, to our knowledge, mechanisms related to the clinical implementation of the GWG have been poorly investigated.

This descriptive qualitative study analyzes the experience of a specialized palliative care unit (PCU) trained in using the GWG, aiming to identify the mechanisms of its clinical implementation and evaluate the tool’s value in personalizing future care.

## 2. Materials and Methods

We chose a descriptive qualitative study [14] by means of a Focus Group (FG), aimed at collecting and comparing the experiences of HPs involved in GWG administration. It was guided by the following research question:

What are the mechanisms that promote or hinder the application of the GWG in clinical practice?

### 2.1. The GWG

The GWG is a card sorting game, initially created in 2005 by Coda Alliance [10]. Its purpose is to facilitate comfortable conversations about difficult end-of-life topics, allowing individuals to make choices about their lives and end-of-life preferences. The original English version includes 35 cards and one “wild card”, each presenting a brief statement on issues relevant to those facing life-threatening illnesses, such as symptom management, personal autonomy, communication with family, and practical daily needs [11]. Players are encouraged to prioritize their wishes as they sort through the cards, identifying the most important desires to discuss with family members or healthcare professionals. These identified wishes and values can then be integrated into a personalized therapeutic plan, enabling the patient to engage in ACP [6].

### 2.2. Setting

The PCU is a specialized hospital-based unit without dedicated beds at Reggio Emilia Hospital, a 900-bed cancer research Italian hospital, accredited as a Clinical Cancer Institute. It is composed of six HPs: three clinicians and three nurses. The PCU assists outpatients and inpatients with advanced oncological or chronic progressive diseases—above all, neurological and respiratory diseases. Its mission is to perform clinical, training, and research activities in PC. In 2024, the PCU conducted 2650 consultations, including 647 initial consultations and 219 family conferences. The PCU’s penetrance, the percentage of cancer patients assessed by the PCU out of the total number of cancer patients hospitalized per year, was 14% [15]. The PCU managed 28% of oncological and non-oncological patients in the “Stable phase” of illness, 50% in the “Unstable phase”, 19% in the “Deteriorating phase”, and 3% of patients in the “Dying phase” of illness [15]. 

### 2.3. Participants

All the PCU’s health care professionals were invited to participate to the FG. FG participants were informed by M.P., a researcher in medical ethics at the Bioethics Unit (BU) of the same Local Health Authority (LHA) with experience in qualitative research, about objectives and participation requirements. The participants were recruited via email by M.P., who ensured participant confidentiality. The HPs involved were specialists in palliative care who had prior training in the use of the GWG and ACP. The training was conducted by the BU’s researchers, from 2021, and consisted of didactic lessons, where the tool was presented and discussed, and role-plays, finalized to experience the personal impact of the GWG and to exercise how to present, propose, and play the tool with patients. In addition, participants involved in the study related to the Italian validation and cultural adaptation of the GWG [12], which also provided them with additional opportunities to deal with the GWG.

### 2.4. Data Collection

Three weeks before the FG, participants were encouraged by M.P. to retrospectively collect the cases in which they applied the GWG in the last three years (from 2021 to May 2024). From 2021, the PCU, in collaboration with researchers form the BU, started working on the GWG, from their involvement in a research project about the Italian validation and cultural adaptation of the tool [12], to its application in clinical practice. HPs were asked to systemically check their consultation with patients, and to report all the cases where they applied the GWG.

By reviewing the consultations conducted, the HPs reflected on the rationale behind the GWG proposal and carefully considered both the instrument and its game modalities. After three weeks, a Focus Group (FG) was organized to discuss and compare data obtained from individual cases [16,17].

The date and time of the meeting were agreed upon with the facilitator and were compatible with the practice obligations of the FG participants. The FG was held in a meeting room at the HPs’ workplace and was audio-recorded. All the participants provided oral consent to be audio-recorded. The facilitator presented and guided the discussion and participant interactions concerning themes and personal opinions.

The FG was divided into sections covering clinical cases, HPs’ perspectives, and the resulting impact on the patient–professional relationship. The interaction between participants was simulated using guiding questions (Table 1).

### 2.5. Data Analysis

We followed the thematic analysis by Braun and Clarke [18]. The FG was transcribed verbatim and analyzed using line-by-line coding, prioritizing participants’ words to maintain authenticity and ensure the model’s originality. The analyses involved two researchers (M.P. and S.A.) who independently analyzed the FG transcript by repeatedly reading the text, extrapolating the themes that emerged and grouping and/or dividing them into categories of content. An iterative process was used to verify the consistency of themes and categories with the transcript, identifying significant sentences that condensed and represented the meaning of the themes and the identified categories. As the analysis proceeded, the researchers were able to combine an inductive approach (in which themes and categories are derived from the data, i.e., from the transcripts) with a deductive approach (in which the categorization process is structured based on the themes and categories of content identified from time to time). Discrepancies between the researchers’ categorizations were resolved through discussion, leading to a final, agreed-upon categorization. Data were presented by reporting participants’ quotations. Every quotation was identified by a code, representing the participant speaking and the related number of the meaning unit.

Subsequently, the researchers systematized the FG’s findings by identifying three phases of the GWG (before, during, and after). For each phase, the team has identified the essential components and their respective barriers and facilitators, with the aim of providing clear indications on the application of the tool in clinical practice.

### 2.6. Rigor

We adhered to Braun and Clarke criteria of credibility, originality, resonance, and usefulness to ensure this study’s rigor [18]. An external reviewer from our team (S.T.) audited the transcripts and codes to provide additional scrutiny. Reflexive practices were integral throughout the research process; researchers maintained reflexive memos to document their positionality and its potential influence on data interpretation. These memos were shared and discussed with an external colleague (S.S. and L.D.P.), promoting critical dialogue and ensuring consistency in analytical rigor. This process strengthened the study’s trustworthiness.

Study procedures and reports followed the Consolidated Criteria for Reporting Qualitative Research (CoreQ) guidelines [19].

## 3. Results

A total of five HPs were involved in the FG: three palliative care physicians and two palliative care nurses. One palliative care nurse could not attend the FG due to illness. Participant professional characteristics are described in Table 2. The FG session lasted approximately 120 min.

At the beginning of the FG, each participant was asked to indicate the total number of cases collected. The HPs reported their experiences using the GWG on 15 patients managed by the PCU within the last three years (2021–May 2024). Patient characteristics are described in Table 3. Comprehensively, the cases reported by the HPs included nine oncological patients, five patients with Amyotrophic Lateral Sclerosis (ALS), and one patient with end-stage cardiopathy.

The analysis of the FG led us to identify five overarching themes with different meanings, defined within their subthemes: (1) personalizing the GWG proposal; (2) the role of the caregiver; (3) organizational aspects; (4) the meaning of the GWG in clinical practice, and (5) dealing with patient’s priorities.

### 3.1. Personalizing the GWG Proposal to the Patient

HPs extensively focused on the proposal of the GWG to their patients.

#### 3.1.1. Different Reasons to Propose the GWG

Participants articulated different reasons and motivations which triggered them to propose the GWG.

- Physical reasons

One physician employed the GWG to enhance communication with a patient experiencing dysarthria, reporting “*I wanted the patient to have guidance towards her priorities in a comfortable way… overcoming a significant physical limitation*” (*COD.1.6-8 Ph*).

- Clinical and decisional uncertainty

In the context of oncological patients, HPs proposed the GWG as a tool to aid patients in clarifying and comprehending their care priorities, especially in instances of decisional uncertainty.

“*… on another patient, an oncological patient, I used the game because he was struggling organizing his priorities. He was a patient with lung cancer, and he was having troubles in choosing what to do*”.

(*COD. 1.9 Ph*)

“*…We suggested the card game to help her understand what mattered most. We said: ‘with these cards, we can explore your values –what’s truly important—during this difficult time’. But she wept, ‘No, I don’t want this…I don’t want die. I want to see my grandchildren again.’ She cried and cried…we had truly reached an impasse*”.

(*COD. 2.60 Ph*)

- Clarify ideas

In other occasions, HPs employed the GWG within ACP to ensure comprehensive exploration of preferences, even with patients exhibiting high self-determination and illness awareness or cases of patients who had already shown opinions around their care. The game served as a structured prompt there, facilitating in-depth illustration of themes revealed by the cards, optimizing the process by minimizing oversight. The GWG was helpful for “*putting in order the thoughts they already have in their head*” (*COD.4.93 Ph*).

- Facilitating the patient’s narration

HPs found the GWG appropriate when they sought to gain insight into a patient’s values and personality, particularly during care planning discussions. In some cases, the tool has been proposed to guide the conversation with the patient, helping them to focus the narration of themselves in the light of a future ACP process.

“*She was a patient who had asked herself for palliative care from the very beginning and she was for a shared care plan, asking about scenarios and whatnot…I started from the GoWish to leave nothing out of the planning…*”.

(*COD.1.11 Ph*)

*One HP offered it to a patient who struggled to speak during the visit, due to personal characteristics. The GWG* “*helped him to have words for an interview*”.

(*COD.4.106 Ph*)

#### 3.1.2. Preconditions for Proposing the GWG

Participants identified some necessary preconditions for applying the GWG to their patients, stressing the importance of an appropriate evaluation of patients’ clinical condition, awareness, a strong relationship of care and HPs’confidence with the tool.

- Controlled symptoms

Participants identified preconditions related to patient characteristics, circumstances, and conditions, as well as the specific actions HPs take within the care relationship. For example, participants agreed on the necessity for patients’ symptoms to be controlled and for patients to be free from depression, anxiety, and fears.

- The patient’s awareness

HPs agreed that the GWG should be proposed only when there was certainty regarding the patient’s awareness of their illness and its life-limiting nature, or when the patient inquired about their prognosis; they also used it when patients had already expressed decisions to pursue treatment planning or avoid therapeutic obstinacy, sometimes by a living will. Conversely, HPs did not propose the tool if they were uncertain about the patients’ desire to think about future care.

“*…there have been previously 2–3 meetings to check if he really wished to work on this issue or if it was just things he said during the visit…*”.

(*COD. 1.28 Ph*)

“*…I noticed that I used it on a lot of them who had Advanced Directives because they were giving me those little clues …*”.

(*COD. 4.104 Ph*)

Conversely, the absence of preliminary patient reflection on desired healthcare treatments hindered HPs from proposing the GWG, as reported by a participant.

“*I thought (the GWG) could help understand her values…what really matters to her, how she sees herself, maybe explore some more concrete scenarios. But then I wondered…is she even asking for this? She came in talking about completely different things. At the moment, proposing the game would have been…inappropriate*”.

(*COD 2.156 Ph*)

- Strong relationship of care

HPs described the experience of proposing the GWG activating the patient emotionally greatly. Participants emphasized the need for careful observation of patient requests, to determine the appropriateness of proposing and “*to select them so as not to hurt them*” (*COD.5.100 Ph*). A strong relationship of care and trust emerged as a common facilitating factor among participants. Finally, all the HPs concurred on the importance of perceiving the patient’s desire to express themselves.

“*…in my opinion, the desire theme is very strong. Now that I’m listening to what you’re saying, I’m associating it with several patients. The need to say certain things to oneself, to a trusted clinician or perhaps to a relative who’s hard to talk with, makes it click: you feel the patient is burning to share something that’s important to them and that’s when you use the tool*”.

(*COD. 5.170 Nurse*)

- HPs self-confidence with the GWG

HPs identified additional facilitating factors, such as self-confidence in dealing with the tool and its impact, as well as the appropriate timing relative to the clinical evolution of the illness: neither too early nor too late. It emerged that the team devoted time to selecting the right moment to propose the game and clearly explaining the game’s goal, as it was not perceived merely as a game. In most cases, a nurse was present.

### 3.2. Role of the Caregiver

Participants extensively discussed the role of the caregiver/family member during GWG meetings and the associated emotional impact also for them. HPs described different situations where patients were either alone or accompanied by a caregiver/family member.

#### 3.2.1. The Presence of the Caregiver

In some cases, it was useful to have the caregiver present during the GWG; in some other cases, the patient decided to play the game alone or the caregiver chose not to participate due to emotional strain.

One HP reported “*The caregiver is not always present, as some (patients) are strongly determined people that might come to the visit alone*” (*COD.1.99 Ph*).

#### 3.2.2. The Impact of the GWG on Caregiver/Family Member Relationship

All HPs acknowledged the emotional impact of the GWG on caregivers/family members. Participants reported how engaging with the game deeply influenced the dynamic between patients and caregivers.

One HP described “*the beauty of the living dynamics among the family members…it was a moment of great contact and emotion …*” (*COD. 2.85 Ph*). However, in other cases, HPs highlighted scenarios where the patient and his familiars were handling the situation with differing emotional attitudes.

“*I remember (the patient) was a very sensitive young man who reflected on the cards. His brother was in great difficulty with the topics that came up…*”

(*COD. 2.40 Ph*)

“*(he) chose cards like ‘die at home’ and ‘not being a burden to my family’ which forced his wife (the caregiver) to confront topics she had avoided. She admitted ‘these cards gave me a lot to process.’ When I mentioned signing Advance Directives at their next pneumo-neuro appointment, she resisted: ’Can we skip this? I don’t even want to come.’ The patient, in the end, chose to attend alone*”.

(*COD. 3.109 Nurse*)

All the HPs reflected upon the possibility that, in some cases, “*…some collateral damage on family members is perceived!*” (*COD. 1.117 Ph*), especially in such relatives who are not ready or willing to discuss the EoL of their loved one. As reported by a participant: “*it’s a fine line to understand how much collateral damage you’ll encounter, and what you decided to accept because it leads to something beneficial. (…) that’s also why we need to ensure these conversations aren’t too far advanced, because then you can’t manage if things go wrong*” (*COD.4.110 Ph*).

Considering the impact of the GWG on care relationships, HPs agreed on the need for continued attention towards caregivers even in subsequent visits following the game.

### 3.3. Organizational Aspects

Reflecting upon their cases, HPs identified some organizational aspects which facilitate the use of the GWG with their patients. First of all, the outpatient setting was considered adequate and favorable for the discussion. Other aspects were to consider the patient’s timelines and game methods and organize multiple meetings in which to deal with themes by theme chosen through the cards.

“*I also split up the cards, when I realize there’s too much going on, and they don’t have a clear understanding of what their values are yet, you risk repeating past mistakes…that is, when you don’t use the cards you might talk about too many things and in the end the message doesn’t come across clearly…*”.

(*COD.1.116 Ph*)

Allowing the patient multiple meetings and appropriate time to reflect has been recognized as a way to create space for considering the addressed topics or the chosen cards. HPs also noted that they document what emerges during the discussions.

“*Then, the second time, I re-read a summary of the values and explanations they gave me, that is, when they explain the cards, I repeat ‘Did I understand correctly?’ and if there’s space, I link all the possible clinical scenarios, which doesn’t necessarily mean making a decision immediately*”.

(*COD.4.188 Ph*)

Referring to a negative experience, two HPs reported that the presence of many professionals in the room, and the ‘urgency’ of decisions for invasive treatment, led to a negative feeling of “*being forced*” (*COD. 2.67 Ph*), with a resulting negative impact on the patient.

“*At the clinic, she didn’t want to talk about certain things…and with all those people…I could tell she felt forced to decide on the spot. I felt it too. Too many people in the intensive care unit, too much hurry*”.

(*COD.3. 63 Nurse*)

### 3.4. Meaning of the GWG in Clinical Practice

Considering the impact of the tool, HPs agreed that playing the GWG strongly influenced both care relationship and patient decision-making processes.

#### 3.4.1. Impact on Care Relationship

The participants agreed that the GWG has a positive impact on communication when addressing difficult conversations with their patients in advance. They noted that the cards helped them discuss EoL issues or death settings with their patients in advance. However, two HPs felt that the game negatively impacted on relationships when the proposal timing was not appropriate for the patient’s emotional state.

“*The patient was having a hard time, she didn’t know what to choose…I told her that we could stop … but I felt as I was a forcing her and perhaps the moment, the setting and the person who was previously not inclined to talk about some themes …she felt the hurry …*”.

(*COD.3.61 Nurse*)

Overall, participants agreed that the GWG reinforced the care relationship between patients and their clinicians: they found that the GWG helped them to better identify and contextualize patients’ needs, and to organize and define the objectives of meetings and discussions.

“*I mean, it’s obvious that if you sit down and ask ‘What are your priorities and values?’, half of them, in my opinion, won’t understand the question… It (the game) also makes your job easier to understand (quickly) what kind of people you have in front of you, what the dynamics are between them and their family members are… so in my opinion, well, this (the game) helped me…*”.

(*COD.1.144 Ph*)

#### 3.4.2. Going Beyond Healthcare Choices During the Decision-Making Process

HPs confirmed that the GWG appears to be a tool that facilitates healthcare choices by helping patients clarify their ideas. However, in one case, it amplified the patient’s confusion and distress. Consequently, HPs stressed that “*it’s not a tool for everyone*” (*COD. 1.179/COD.4.197Ph*).

- Explore many more aspects of care

When applied to highly self-determined patients, HPs noted that the game allowed them and their patients to move beyond healthcare choices regarding treatments and to explore many more aspects of care and/or care interventions on the person, especially in regard to family relationships and patient priorities around them. For instance, one HP reflected that the tool helped her change her attitude towards ACP with oncology patients, prompting her to propose ACP more frequently to this population.

- Identify family dynamics and vulnerabilities

The GWG allows for the identification of family dynamics and vulnerabilities that would not have otherwise emerged. In one case, applying the GWG provided an “*opportunity for a spiritual intervention*” (*COD. 2.84 Ph*), and in another, it helped patients emphasize relational and affective aspects with family members involved in the discussion.

“*…I sensed the game highlighted a more affective-relational need, a need for reconciliation and recognition. This patient craved acknowledgment from his family: he wanted to make peace with them, with his illness, with his choices, and he wanted them to align with him. All these things, in short, go a bit beyond the tool itself: so he used the game to talk about himself, even with his brother present*”.

(*COD.2.45 Ph*)

- Clarify the trustee’s role

The GWG discussion sometimes revealed the necessity of clarifying the caregiver/trustee’s role, “*That is somewhat functional in helping the trustee or caregiver understand what it means to be a trustee*” (*COD. 4.89 Ph*), namely, the individual who will represent the patient’s wishes in the event of cognitive decline. In one instance, the patient revised their trustee designation following the GWG discussion, selecting their physician: “*I found the GWG strengthened my relationship with the patient because I was the custodian of what they said to me, but at the same time, I was the messenger between the patient and his family*” (*COD. 1.122 Ph*).

### 3.5. Dealing with the Patient’s Priorities

During the FG, HPs reflected on what happened during the GWG game discussions. They identified the most recurring themes shared with patients through the GWG, which are reported in Table 4. Specifically, HPs reported greater ease in dealing with the topic of palliative sedation.

The dignity card was acknowledged by all the participants as highly functional, not only for making decisions but also for gaining a deeper understanding of the individuals and their care needs. It was described as “*Very functional, particularly in helping caregivers understand what constitutes dignity for a loved one*” (*COD. 5.62 Nurse*).

In addition, all participants highlighted two different dynamics which occasionally complicate the decision-making process: conflicts within patients’ priorities and situations when the patients’ card choices differ from HPs’ expectations.

#### 3.5.1. The GWG Revealed Conflicts Within Patients’ Priorities

According to one HP, the tool gives patients the opportunity to mull over several aspects of their care which may reflect diverse needs and expectations. It has been defined as a “*non-linear process*” (*COD.1-2-5.73/74*) around which they may find it challenging to offer solutions.

“*He said he picked the family card up: avoiding arguments, not being a burden, preparing the family, and then at the same time he said he wanted to die at home. There were conflicts among the things he brought up*”.

(*COD.2.42 Ph*)

#### 3.5.2. The Patient’s Card Choices Differ from HP’s Expectations

In a few cases, this happened: “*sometimes it goes as I imagined it, sometimes it doesn’t. Proof the expectation you have isn’t aligned with what comes out eventually. My needs might be some things, but the patients’ needs are sometimes different*” (*COD.1.135 Ph*).

HPs shared their feeling of surprise when patients chose or excluded cards that, from the HP’s point of view, could be relevant to their condition, such as “not being dependent on a machine”, in the case of an ALS patient. Focusing on such a situation, HPs agreed that patients in these cases choose cards on topics they have never had a chance to discuss with anyone, or cards reflecting questions they didn’t have answers to. HPs highlighted the risks of interpreting a patient’s priority and redefined “patient’s priority” as something concretely embedded in the patient’s experience.

“*This thing about priority comes up often, (…) the eliminated cards are important, but they represent aspects the patient is addressing or has resolved already. So, priority in this context is not necessarily value-based. While values are part of it, the focus is on what is closest to their hearts. We might call these values, but for them, it could also include finances, which are a value too*”.

(*COD.1-2.139*)

#### 3.5.3. Comparative Analysis and Practical Implications

Based on the FG’s findings, comparative analysis of cases involving real-world applications of the GWG has led to the identification of several components that may either impede or facilitate the utilization of the tool in alignment with personalized care. These components, along with their associated facilitators and barriers, are presented in Table 5.

## 4. Discussion

This descriptive qualitative study aims to identify the active mechanisms, barriers, and facilitators of GWG in clinical practice with patients affected by life-limiting illnesses.

Compared with previous research [13], our findings provide meaningful insight into the practical application of the GWG in clinical practice, as experienced by a group of specialized palliative care HPs trained in the use of the GWG, finally identifying several components and related concrete strategies to improve an appropriate and personalized application of the tool.

Key findings from our study revealed that the GWG represents an appropriate and useful tool in helping patients articulate thoughts, feelings, and personal narration, aiding the patient in the challenging process of deciphering and prioritizing values [6,13,20,21]. It also emerged that the GWG is not suitable for every patient at the end of life, especially those who are not aware of their condition or ready to discuss EoL care. Furthermore, our data showed that several components should be carefully evaluated and prepared by the HPs involved in each of the three phases of GWG application (before, during, and after the GWG).

First of all, preparing a meaningful and well-supported end-of-life discussion by means of the GWG requires a broader attention to symptom management and timing of the GWG proposal. It emerged how the most congenial time for proposing the game to the patients is not relative to illness, prognosis, or urgency in making medical decisions, but rather to them and their family members being ready to face certain topics, and to the depth of their relationship with the healthcare team and the clinician who decides to “get in the game”. The quality of the healthcare relationship and collaborative teamwork are pivotal elements to ensure a tailored application of the GWG to the patient and caregivers. That reinforces the significance of a multidisciplinary approach and the central role of the patient, both of which are key aspects of the palliative care approach, and underscores why addressing these issues within a long-lasting care relationship is more effective than in an urgent care setting [22,23].

Another interesting finding concerns the potential “collateral damage” associated with the use of the GWG, especially regarding the emotional impact on the caregivers and on the HPs. Sometimes using the GWG leads to emerging challenges between the patient and their loved ones: despite the emotive difficulties to deal with it, it also represents an important opportunity to go deeper into familiar dynamics and to reflect upon the role of the caregiver along a subsequent ACP [13]. As part of the palliative care approach, caregiver involvement should not be taken for granted [24]: a deep knowledge of the dyad allows for the identification of potential collateral damage that the game could cause and the discovery of new strategies and resources to deal with it. Giving particular attention into preparing the caregiver for what will be shared and discussed during the GWG facilitates shared decision-making with the healthcare team and loved ones, and also the ACP process and the trustee identification [25].

Our findings also describe detailed practical and organizational actions to personalize a GWG discussion, concerning the setting, meetings, and personnel involved.

Our findings described the outpatient setting as the more promising for GWG application. In other experiences, the GWG appears particularly beneficial for hospitalized patients undergoing prolonged infusion treatments or experiencing long waiting intervals between consultations [26]. Identifying the best setting for the GWG remains an open question, but carefully selecting and preparing the setting for the GWG, taking into account patient privacy and the specific clinical needs of inpatients, should be always considered [27], as also emphasized by the experiences of our participants.

Another unresolved subject is the method of GWG administration: the data do not confirm whether it should be administered in the presence of a professional. If so, the type of professional (physician and/or nurse) and the nature of their interaction with the patient remain unclear [13]. The study, however, highlights the importance of the sheer number of people attending and observing the GWG, especially when it is used on the most fragile patients, like hospitalized ones. As a key point, the significance of the presence of clinicians who are the main point of reference for the patient is evident.

Additionally, it is not possible to quantify the necessary time and number of meetings for GWG. It is plausible that standardization is difficult due to the high level of personalization required by a tool that explores very personal issues and is highly dependent on context-specific and HP-specific factors as well. Providing evidence on these aspects may be helpful in dealing with workload burden, lack of time and inadequate support, which are frequently cited as GWG barriers [13,28].

Finally, it must be noted that several skills and competencies, such as empathy, compassion, active listening, and effective communication, are essential to create the appropriate environment where the GWG may be applied [29]. These skills, along with other ethical and relational competencies, may also be pivotal to overcome the barriers and difficulties concerning the GWG, such as dealing with patients’ conflicting priorities.

Participants in our study were highly specialized in palliative care [12,30,31], holding ethical, communicative, and relational competencies, and in GWG application as well. It suggests that applying the GWG requires additional training for HPs, who should develop specific practical skills on GWG application during intensive, dedicated training [28]. Few studies exist regarding training in GWG application: training on the GWG may provide HPs with the ability to establish specific game modalities to limit collateral damages that might arise, and to deepen the ethical and relational skills to deal with EoL discussion. Further studies are required to confirm the competencies and skills required to implement the GWG and the appropriate training structure to develop them. It has already been shown that serious educational games are effective in improving various health outcomes and skills, among which is decision-making [32]. Then, as it is a serious educational game, GWG training may be integrated into ACP intervention, tailoring healthcare teams wishing to start ACP—or treating patients potentially involved in ACP—in a variety of care settings, not only in palliative care (as we developed with the following research project in oncological and hematological setting ClinicalTrials.gov Identifier NCT06795815).

### Study Limitations

The most important limitation of this study is the experience of one single center and the characteristics of the participants involved, which may impact the reproducibility of the findings. The involvement of professionals who are not trained in the tool’s use could lead to different outcomes. Therefore, our study focuses on the experiences of trained professionals to provide insights into the tool’s applicability in clinical practice, which may be useful for other HPs who seek guidance and training on the application of the GWG. Due to data confidentiality, is not possible to better describe all 15 cases.

## 5. Conclusions

The study arose from the need to identify a model for implementing the GWG in clinical practice. Our findings describe differences and patterns among palliative care specialists working within the same PCU who applied the GWG with their patients. While some patterns may be identified, certain aspects still require attention, particularly regarding the training and competencies (communicative, relational, ethical) of the healthcare professionals, and the process leading to the proposal of the GWG to the patient. Moreover, further studies are needed to understand patients’ and caregivers’ perspectives about the whole GWG process, to keep personalizing the intervention, and to adapt it to the settings of both oncological and other chronic progressive diseases.

Altogether, our data highlight how an intervention of this type develops on multiple levels and therefore can be considered as a complex intervention [33]. These levels include aspects related to the ethical, relational, and communicative skills of the operators, as well as the setting and time allocated for sustainable and effective implementation. Further research is necessary to fully elucidate the implications of its application.

## Figures and Tables

**Table 1:** Focus group guiding questions.

Phase	Guide Questions
(1) Story-telling	- Tell me some significant cases in which you used the GWG. - Describe some features of the patient. - Who conducted the game? - Who was present? - How was the tool presented and what critical issues did you meet? - How was the proposal welcomed? - How did the meeting take place? - How did it end? - Were the family members present? If not, why? - Did the game facilitate the ACP?
(2) HPs’ perception	- How did you feel when proposing the GWG? - How did you feel while conducting the GWG? - What reflections were there after the GWG?
(3) Impact on HPs	- What did you get from GWG? - What did you hope to get? - Could something go differently? - What were the differences between your expectations and what you achieved?

**Table 2:** Characteristics of the FG participants.

Code	HPs Features	Years of Experience in PC
1	Palliative Care Physician	13 years
2	Palliative Care Physician	7 years
3	Palliative Care Nurse	13 years
4	Palliative Care Physician	15 years
5	Palliative Care Nurse	9 years

**Table 3:** Patient characteristics.

Diagnosis	
Cancer	9/15
Metastatic lung cancer Metastatic pancreatic cancer Metastatic prostate cancer Hepatic carcinoma Metastatic ovarian cancer	3/15 2/15 2/15 1/15 1/15
Amyotrophic Lateral Sclerosis	5/15
End-stage Cardiopathy	1/15
Average days in charge	
Median	12 months
Range	24 days–48 months
GWG setting	
Outpatients	14/15
Inpatients	1/15
Presence of caregiver	9/15

**Table 4:** Topical topics.

Topics	Significant Quotation
Palliative sedation	“*Sedation. That always comes up because there are many questions that lead you back there, that is, many phrases that lead you back there when they choose (the card)*” (*COD.1.29 Ph*).
Dignity	“*The dignity card is also very functional for caregivers to help them understand what is dignified for their loved ones*” (*COD. 4.96 Ph*).
- Family: - “Don’t be a weight for my family” - Importance and challenges of family members	“*… these are medical choices, but rather choices about the setting. The setting, as far as I’m concerned, comes frequently up in oncology, just like the involvement of family members does*” (*COD.2.82 Ph*).
Advance Care Planning - Choice of treatments - Choice of trustee - Choice of death setting	“*With ALS patients, obviously, these conversations are gold, because from there it stems the choices about: tracheostomy, PEG, or breathing support (NIV)*” (*COD.1.32 Ph*).
Humor	“*in my experience too, there’s a lot of dignity card, there’s a lot of humor card too*” (*COD. 4.129 Ph*).

**Table 5:** Descriptions of the components involved in a personalized application of the GWG *.

Phase of the Intervention	Component	Facilitator	Obstacle
Before the GW	*Assessing the most favorable condition during the relationship of care*	- Ensuring symptom control - Being certain of patients’ awareness of their illness and life-limiting disease - Careful observation of patient’s requests (i.e., patient’s questions about prognosis) - Appropriate timing for implementation - Self-confidence in using the tool - A trust relationship with the PCU	- Presence of depression, anxiety, and fears. - Absence of a preliminary reflection by the patient on what he/she would receive in terms of healthcare treatments - Uncertainty about the patient’s will to think about future care - ‘urgence’ of making the decision about care
	*Patient-related factors*	- Self-determined patients - Patients aware of their illness - Patients who have already expressed opinions about their care	- Patients with much fear and confusion
	*A personalized purpose to apply the GW*	- Feel the need to know the person, his/her values and personality - Compensates for physical disabilities (e.g., dysarthria) - Clarify and understand care priorities - Addresses patient questions about prognosis and future care - Guides conversation during Advance Care Planning (ACP)	- An excessively rigid instrument that may constrain the patient’s self-narrative - The necessity to apply an innovative EoL conversation tool
During the GW	*Caregiver/family-related factors*	- Dedicating time to deal with the emotional impact of the GW on the caregiver/family member	- Caregivers/family members not ready or willing to discuss end-of-life care
	*Setting and approach*	- Outpatient setting - Multiple meetings - Consideration of patient’s timelines	- Multiple professionals involved - Adapting to various healthcare settings (e.g., hospital vs. outpatient) - Inappropriate timing for GW implementation
	*HP-patient discussion*	- Discovering different interpretation of patient’s priorities	- Difficult discussion when patient’s priorities collide
After the GW	*Caregiver/family-related factors*	- Need for ongoing attention to caregivers in subsequent visits	- Dealing with the emotional impact of GW on the caregiver/family member
	*ACP and healthcare choices*	- Reinforcing previous healthcare choices - Going beyond healthcare choices and discussing other EoL issues (setting, trustee…) - Reflecting on patient-family relationship	- Being too focused on formalizing choices within ACP

* The three different background colors are associated with the three phases of the GWG.

## Data Availability

The original contributions presented in this study are included in the article. Further inquiries can be directed to the corresponding author(s). The raw data supporting the conclusions of this article will be made available by the authors on request.

## References

[1] Sudore R.L., Lum H.D., You J.J., Hanson L.C., Meier D.E., Pantilat S.Z., Matlock D.D., Rietjens J.A.C., Korfage I.J., Ritchie C.S. (2017). Defining Advance Care Planning for Adults: A Consensus Definition From a Multidisciplinary Delphi Panel. J. Pain Symptom Manag..

[2] Knight K. (2021). 50 Years of Advance Care Planning: What Do We Call Success?. Monash Bioeth. Rev..

[3] Bernacki R.E., Block S.D. (2014). Communication about Serious Illness Care Goals: A Review and Synthesis of Best Practices. JAMA Intern. Med..

[4] Jimenez G., Tan W.S., Virk A.K., Low C.K., Car J., Ho A.H.Y. (2018). Overview of Systematic Reviews of Advance Care Planning: Summary of Evidence and Global Lessons. J. Pain Symptom Manag..

[5] Rietjens J.A.C., Sudore R.L., Connolly M., van Delden J.J., Drickamer M.A., Droger M., van der Heide A., Heyland D.K., Houttekier D., Janssen D.J.A. (2017). Definition and Recommendations for Advance Care Planning: An International Consensus Supported by the European Association for Palliative Care. Lancet Oncol..

[6] Lankarani-Fard A., Knapp H., Lorenz K.A., Golden J.F., Taylor A., Feld J.E., Shugarman L.R., Malloy D., Menkin E.S., Asch S.M. (2010). Feasibility of Discussing End-of-Life Care Goals with Inpatients Using a Structured, Conversational Approach: The Go Wish Card Game. J. Pain Symptom Manag..

[7] Hasegawa T., Okuyama T., Akechi T. (2024). The trajectory of prognostic cognition in patients with advanced cancer: Is the traditional advance care planning approach desirable for patients?. Jpn. J. Clin. Oncol..

[8] Fahner J.C., Beunders A.J.M., van der Heide A., Rietjens  J.A.C., Vanderschuren M.M., van Delden J.J.M., Kars RN M.C. (2019). Interventions Guiding Advance Care Planning Conversations: A Systematic Review. J. Am. Med. Dir. Assoc..

[9] De Vleminck A., Houttekier D., Pardon K., Deschepper R., Van Audenhove C., Vander Stichele R., Deliens L. (2013). Barriers and Facilitators for General Practitioners to Engage in Advance Care Planning: A Systematic Review. Scand. J. Prim. Health Care.

[10] Coda Alliance Website. https://codaalliance.org/.

[11] Menkin E.S. (2007). Go Wish: A Tool for End-of-Life Care Conversations. J. Palliat. Med..

[12] Perin M., Tanzi S., Botrugno C., Craddock C., Menkin E., Peruselli C., De Panfilis L. (2022). Translation and Cultural Adaptation of the *Go Wish Game*: Thinking About Personal Values to Promote Advance Care Planning. J. Palliat. Med..

[13] Paiva B.S.R., Trevizan F.B., de Oliveira L.C., da Costa Rosa K.S., Betussi V.A., Lourenço B.M., Julião M., Paiva C.E. (2025). Go Wish Card Game for Meaningful Conversations in the Oncology Healthcare Context: A Narrative Review. Cancers.

[14] Holloway I. (2013). Qualitative Research in Nursing and Healthcare.

[15] Alquati S., Peruselli C., Turrà C., Tanzi S. (2022). Lesson Learned From Hospital Palliative Care Service in a Cancer Research Center in Italy: Results of 5 Years of Experience. Front. Oncol..

[16] Holloway I., Galvin K. (2016). Qualitative Research in Nursing and Healthcare.

[17] Kitzinger J. (1995). Qualitative Research. Introducing Focus Groups. BMJ.

[18] Braun V., Clarke V. (2019). Reflecting on reflexive thematic analysis. Qual. Res. Sport Exerc. Health.

[19] Tong A., Sainsbury P., Craig J. (2007). Consolidated Criteria for Reporting Qualitative Research (COREQ): A 32-Item Checklist for Interviews and Focus Groups. Int. J. Qual. Health Care.

[20] Pazart L., Vidal C., Chalon D.F., Gauthier S., Schepens F., Cretin E., Beal J.-L., Pfitzenmeyer P., Aubry R. (2011). “Card Sorting”: A Tool for Research in Ethics on Treatment Decision-Making at the End of Life in Alzheimer Patients with a Life Threatening Complication. BMC Palliat. Care.

[21] Tishelman C., Eneslätt M., Menkin E., Lindqvist O. (2022). Developing and Using a Structured, Conversation-Based Intervention for Clarifying Values and Preferences for End-of-Life in the Advance Care Planning-Naïve Swedish Context: Action Research within the DöBra Research Program. Death Stud..

[22] Hui D., Bruera E. (2016). Integrating palliative care into the trajectory of cancer care. Nat. Rev. Clin. Oncol..

[23] Zimmermann C., Mathews J. (2022). Palliative Care Is the Umbrella, Not the Rain-A Metaphor to Guide Conversations in Advanced Cancer. JAMA Oncol..

[24] Song M.K., Manatunga A., Plantinga L., Metzger M., Kshirsagar A.V., Lea J., Abdel-Rahman E.M., Jhamb M., Wu E., Englert J. (2024). Effectiveness of an Advance Care Planning Intervention in Adults Receiving Dialysis and Their Families: A Cluster Randomized Clinical Trial. JAMA Netw. Open.

[25] Wagner C.D., Johns S., Brown L.F., Hanna N., Bigatti S.M. (2016). Acceptability and Feasibility of a Meaning-Based Intervention for Patients With Advanced Cancer and Their Spouses: A Pilot Study. Am. J. Hosp. Palliat. Care.

[26] Azizoddin D.R., Thomas T.H. (2022). Game Changer: Is Palliative Care Ready for Games?. JCO Clin. Cancer Inform..

[27] Batchelor F., Hwang K., Haralambous B., Fearn M., Mackell P., Nolte L., Detering K. (2019). Facilitators and barriers to advance care planning implementation in Australian aged care settings: A systematic review and thematic analysis. Australas. J. Ageing.

[28] Goswami P. (2023). Impact of Advance Care Planning and End-of-Life Conversations on Patients with Cancer: An Integrative Review of Literature. J. Nurs. Scholarsh..

[29] Paiva B.S.R., Mingardi M., de Almeida L.F., de Camargos M.G., de Oliveira Valentino T.C., Julião M., Paiva C.E. (2024). Go Wish Card Game-Exploring End-of-Life Wishes of Patients in Oncology Palliative Care: A Qualitative Study. Ann. Palliat. Med..

[30] Perin M., Tanzi S., Peruselli C., De Panfilis L. (2023). Insegnare Il Go Wish Game: Un Training per l’utilizzo Del Go Wish Con Professionisti Sanitari. Riv. Ital. Cure Palliat..

[31] Tanzi S., De Panfilis L., Costantini M., Artioli G., Alquati S., Di Leo S. (2020). Development and preliminary evaluation of a communication skills training programme for hospital physicians by a specialized palliative care service: The ‘Teach to Talk’ programme. BMC Med. Educ..

[32] Sharifzadeh N., Kharrazi H., Nazari E., Tabesh H., Edalati Khodabandeh M., Heidari S., Tara M. (2020). Health Education Serious Games Targeting Health Care Providers, Patients, and Public Health Users: Scoping Review. JMIR Serious Games.

[33] Skivington K., Matthews L., Simpson S.A., Craig P., Baird J., Blazeby J.M., Boyd K.A., Craig N., French D.P., McIntosh E. (2021). A New Framework for Developing and Evaluating Complex Interventions: Update of Medical Research Council Guidance. BMJ.

